# Position Detection Method of Piezoelectric Driven Spherical Motor Based on Laser Detection

**DOI:** 10.3390/mi13050662

**Published:** 2022-04-23

**Authors:** Zheng Li, Yiding Zhu, Bo Xie, Ye Wang, Xiaoqiang Guo, Hexu Sun

**Affiliations:** 1 School of Electrical Engineering, Hebei University of Science and Technology, Shijiazhuang 050018, China; zhuyiding@stu.hebust.edu.cn (Y.Z.); xiebo@stu.hebust.edu.cn (B.X.); wy19812005@163.com (Y.W.); 2 School of Electrical Engineering, Yanshan University, Qinhuangdao 066004, China; gxq@ysu.edu.cn

**Keywords:** position detection, Doppler effect, laser measurement, multi-DOF spherical motor

## Abstract

Laser detection technology has manypromising applications in the field of motor speed and position measurement. Accurate and fast measurement of position information of spherical rotor is very important for motor control. In this paper, we propose a method for non-contact measurement of the angular velocity of a multi-DOF spherical motor using the Doppler effect of the laser, and further obtain the position information of the motor rotor. The horizontal laser beam from the laser generator is divided into a reference beam *I* and a measurement beam *II* through a beam splitter, and the measurement beam *II* reflects and undergoes Doppler effect after irradiating the rotating motor. The two beams pass through the photoelectric conversion module to obtain the corresponding frequency difference signals to derive the angular velocity and position information of the motor rotor. The correctness of the method is verified experimentally. The results show that the coordinate error of *Z* and *Y* axes is less than 2 mm, thatthe error of *Z*-axes is less than 0.2 mm, and that the method can better measure the spherical rotor position information of the motor.

## 1. Introduction

With the continuous development of modern industrial technology, robotic arms [1] and other devices capable of accomplishing complex spatial motions have been widely used in industrial manufacturing. Traditional multi-degree-of-freedom space motion usually requires the cooperation of multiple motors as well as mechanical actuators. However, the use of actuators causes large system size, low transmission efficiency, and slow dynamic response. To simplify the structure of mechanical systems and improve their dynamic performance and economic efficiency [2], research on spherical motors has been increased in recent years. Various forms of spherical motors such as permanent magnet type [3,4], induction type [5], variable reluctance type [6], and ultrasonic type [7] have been developed one after another. The complex multi-DOF space motion cannot be controlled without the operating system, and fast and accurate rotor position detection is a prerequisite for accurate motor control. The complex structure of the spherical motor makes rotor position detection more difficult to achieve, which has become one of the problems restricting the development and application of the spherical motor. The rotor position detection is divided into contact and non-contact detection according to whether it is connected to the motor rotor or not. The contact detection method usually adds a mechanical connection device between the rotor and the detection mechanism [8,9,10], which has a high detection accuracy, but the use of the mechanical connection device increases friction and inertia moment, and the cost is high and the structure is complicated. The non-contact detection method, on the other hand, has become a research hotspot in recent years sinceit has no direct contact with the motor rotor, has a lower impact on the motor operation, and is more reliable. In the literature [11,12], a MEMS-based detection method for spherical motors was proposed, and the detection of rotor position was achieved by error compensation, but the error parameters of MEMS devices change with time, making the detection require multiple measurements and repetitions to obtain accurate results. In the literature [13], a dual optical sensor was used to acquire images of the continuous variation of the spherical rotor to obtain the rotor position. In [14], a grid map was generated by pseudo-coding and the rotor position was localized using a vision sensor. It is difficult to keep the rotor clean or the painted grid clear after a long period of motor operation, making it difficult to maintain the accuracy of the optical and vision sensor-based detection methods. A method based on linear Hall elements to achieve rotor position detection was proposed in [15], but the Hall elements are susceptible to the rotor magnetic field as well as the geomagnetic field, which causes large measurement errors [16,17].

In this paper, we propose a method of rotor position detection using a laser, which generates the Doppler effect when the laser beam is directed at the moving object. The rotor angular velocity can be measured by calculating and analyzing the reference beam and the measurement beam. Finally, the rotor attitude information can be accurately obtained by integrating the time. The detection method is a non-contact detection method, which avoids direct contact with the rotor and is not affected by the rotor motion and the environmental magnetic field, and has significant advantages such as high sensitivity and fast detection speed.

## 2. Principle of Speed Measurement and Method of Position Detection

### 2.1. Laser Speed Measurement Principle

**Figure 1:**
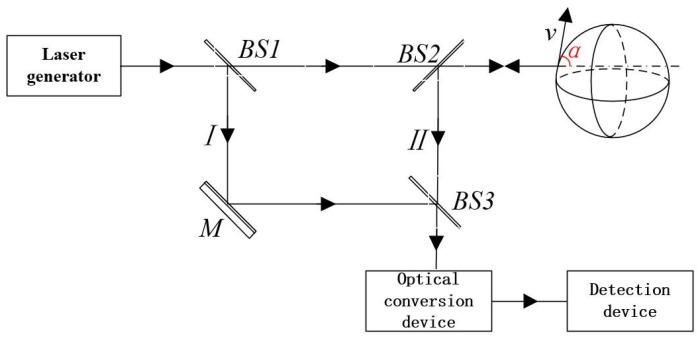
Laser vibration measurement principle.

The schematic diagram of the measurement optical path of the laser vibrometer is shown in Figure 1. The laser beam is divided into a reference beam *I* and a measurement beam *II* through beam splitter *BS1*. The measurement beam *II* is directly irradiated on the measured object and reflected on its surface to produce the Doppler effect frequency shift [18], then guided by beam splitter *BS2* and superimposed on the reference beam *I* at *BS3* to the photoelectric conversion device. The electric field intensity of the two light waves is given by the following equation:

(1) $$E_{r}=E_{r0}e^{i\left(\omega t+\phi_{r}\right)}$$

(2) $$E_{m}=E_{m0}e^{i\left(\omega^{\prime \prime }t+\phi_{m}\right)}$$

where, *E*_r_, *E*_m_ are the electric field intensity of reference beam *I* and measurement beam *II* at the photoelectric conversion device; *E*_r0_, *E*_m0_ are the amplitudes of reference beam *I* and measurement beam *II*; *ω*, *ω*″ are the angular frequencies of light waves; and *ϕ*_r_, *ϕ*_m_ are the phases of reference beam *I* and measurement beam *II* at *t* = 0.

The intensity of light reaching the photoelectric conversion device is:

(3) $$I\propto \left(E_{r}+E_{m}\right){\left(E_{r}+E_{m}\right)}^{*}$$

(4) $$I=I_{r}+I_{m}+2\sqrt{I_{r}I_{m}}\cos\left(\left(\omega^{\prime \prime }-\omega \right)t+\phi_{m}-\phi_{r}\right)$$

where, *I*_r_ and *I*_m_ are the light intensity of the reference beam *I* and the measurement beam *II* at the photoelectric conversion device, respectively. 

The light intensity *I* is a periodic function on the amplitude angle of the cosine function in Equations (3) and (4) with the following range:

(5) $$I_{r}+I_{m}-2\sqrt{I_{r}I_{m}}⩽I⩽I_{r}+I_{m}+2\sqrt{I_{r}I_{m}}$$

**Figure 2:**
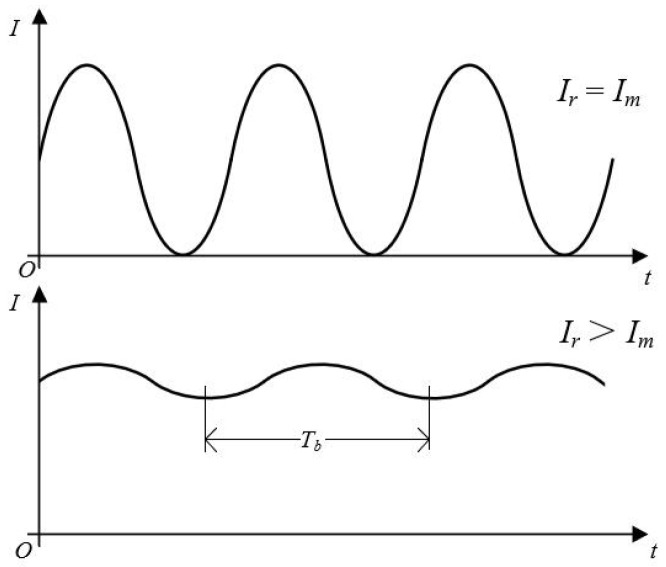
Diagram of light intensity waveform.

The light intensity reaches its maximum when *I*_r_ = *I*_m_. As shown in Figure 2.

The time interval of the peaks in Figure 2 is given by the beat frequency period *T*_b_* = 2π/|ω″ − ω|*, which gives:

(6) $$I=I_{r}+I_{m}+2\sqrt{I_{r}I_{m}}\cos\left[2kv(\cos\alpha )t+\phi_{m}-\phi_{r}\right]$$

Therefore, the beat frequency period becomes:

(7) $$T_{b}=2\pi /|2kv\cos\alpha |$$

The beat frequency period *T*_b_ is measured, and the velocity component of the object in the direction of the measured beam is obtained by Equation (6).

According to Equation (6), it can be seen that the beat frequency period is only related to the velocity gain modulus, and thus the direction of the velocity cannot be distinguished. To solve this problem, the acousto-optic modulator in the reference optical path *I* to achieve this frequency shift, the principle is to reduce the angular frequency of the reference beam by a consistent angular frequency *ω*_0_.

Thus, for reference beam *I*, the following equation holds:

(8) $$E_{r}=E_{r0}e^{i\left(\omega t-\omega_{0}t+\phi_{r}\right)}$$

where, *ω*_0_ is the fixed angular frequency shift of the reference beam.

As shown in Equations (3) and (4), the light intensity of this reference beam can be derived as:

(9) $$I=I_{r}+I_{m}+2\sqrt{I_{r}I_{m}}\cos\left[2kv(\cos\alpha )t+\omega_{0}t+\phi_{m}-\phi_{r}\right]$$

The beat frequency period is:

(10) $$T_{b}=2\pi /\left|2kv\cos\alpha +\omega_{0}\right|$$

**Figure 3:**
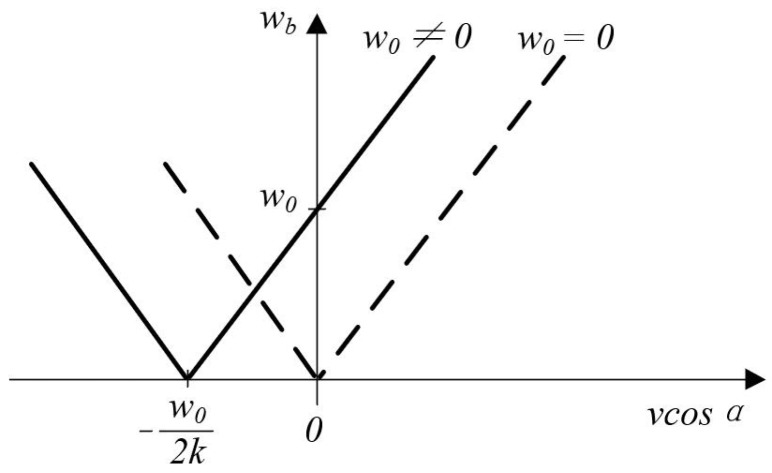
Velocity component and angular beat frequency.

The relationship between velocity component *v*cos*α* and angular frequency *ω*_b_ for the two cases of Equations (7) and (10) is shown in Figure 3. From the figure, it is clear that the direction of velocity cannot be determined for a given beat angular frequency *ω*_b_ when the case of *ω*_0_ = 0. And for the case when *ω*_0_ ≠ 0 it is possible to introduce the direction of velocity.

(11) $$v\cos\alpha >-\omega_{0}/(2k)$$

In practice, the typical value of the cross-frequency shift is *ω*_0_ = 2.5 × 10^8^ rad/s. From Equation (11) and the optical wavelength of the laser beam *λ =* 663 mm, the measurable velocity component in the direction of the measured beam *IIv*cos*α* > −12.7 m/s. Considering the further processing of the angular beat frequency vibration signal, the velocity range that can determine the direction is usually specified as follows:

(12) $$0⩽|v\cos\alpha |⩽\omega_{0}/(2k)$$

The range of angular beat frequencies *ω*_b_ = 2*k* *v*cos*α* + *ω*_0_ derived from Equation (11) is 0 *≤ ω*_b_ ≤ 2*ω*_0_. The signal from the frequency-modulated photoelectric converter is demodulated to produce a signal proportional to the velocity component *v*cos*α*.

### 2.2. Determine the Rotor Position Principle

In the study of position detection of multi-DOF spherical motors, obtaining the exact spatial coordinates of the rotor at a given moment is a prerequisite for motor control technology. In order to get accurate position data, it is necessary to choose to establish a reference coordinate system and a moving coordinate system [19]. This section determines the position detection method of the multi-DOF spherical motor by establishing two coordinate systems and deriving the conversion method between the coordinate systems.

**Figure 4:**
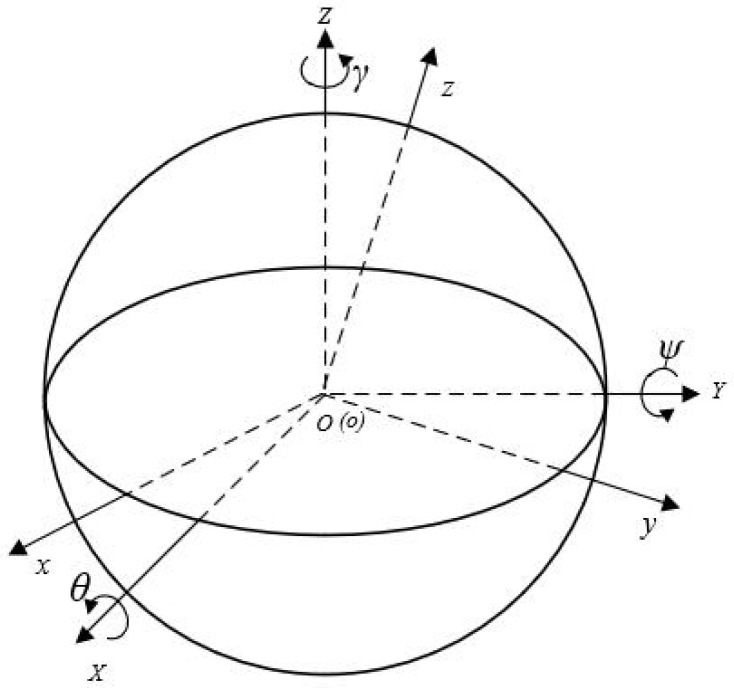
Static and dynamic coordinate systems.

As shown in Figure 4, firstly, the geometric center of the inner cavity of the spherical motor housing is taken as the origin, noted as point *O*, and a reference coordinate system *O-XYZ* is established, where the plane *XOY* is parallel to the horizontal plane and the *Z*-axis is perpendicular to the horizontal plane vertically upward. This coordinate system is used as the reference coordinate system in the position detection system [20,21,22].

As shown in Figure 4, in the initial state of the motor, the dynamic coordinate system *o-xyz* coincides with the reference coordinate system *O-XYZ*, and the three coordinate axes are axially identical. The position of the coordinate system changes with the rotor motion and the *z*-axis is always kept through the top point of the spherical shell.

Due to the special structure of the spherical rotor, the rotor position information can be obtained by determining the angular offset of the spherical rotor at three positions. In which, using the linear xo direction as the laser irradiation direction, the rotor attitude angle is defined as follows:

1. Roll angle *θ*: the angle between the *y*-axis of the dynamic coordinate system *O-xyz* and the *XOY* plane of the reference coordinate system *O-XYZ*, positive when the y-axis is above the *XOY* plane, negative when the opposite is true;
2. Pitch angle *ψ*: the angle between the x-axis of the dynamic coordinate system *o-xyz* and the *XOY* plane of the reference coordinate system *O-XYZ*, when the x-axis is located above the *XOY* plane takes positive, and vice versa takes negative;
3. Yaw angle *γ*: the angle between the x-axis of the dynamic coordinate system *o-xyz* and the *XOZ* plane of the reference coordinate system *O-XYZ*, positive when the x-axis is on the right side of the *XOZ* plane, negative when the opposite is true.

According to this definition, the following rotation matrix is obtained.

When rotating around the *X*-axis, the rotation change matrix is:

(13) $$C_{x}=\left[\begin{array}{ccc} 1 & 0 & 0\\ 0 & \cos\theta & \sin\theta \\ 0 & -\sin\theta & \cos\theta \end{array}\right]$$

When rotating around the *Y*-axis, the rotation change matrix is:

(14) $$C_{y}=\left[\begin{array}{ccc} \cos\psi & 0 & -\sin\psi \\ 0 & 1 & 0\\ \sin\psi & 0 & \cos\psi \end{array}\right]$$

When rotating around the *Z*-axis, the rotation change matrix is:

(15) $$C_{z}=\left[\begin{array}{ccc} \cos\gamma & -\sin\gamma & 0\\ \sin\gamma & \cos\gamma & 0\\ 0 & 0 & 1 \end{array}\right]$$

By changing the rotation of *X*, *Y*, and *Z* axes, the following equation is obtained:

(16) $$\left[\begin{array}{l} X\\ Y\\ Z \end{array}\right]=C_{z}C_{y}C_{x}\left[\begin{array}{l} x\\ y\\ z \end{array}\right]$$

Bringing Equations (13)–(15) into Equation (16), the rotation matrix CR is obtained as:

(17) $$C_{R}=C_{z}C_{y}C_{x}=\left[\begin{array}{ccc} \cos\gamma & -\sin\gamma & 0\\ \sin\gamma & \cos\gamma & 0\\ 0 & 0 & 1 \end{array}\right]\left[\begin{array}{ccc} \cos\psi & 0 & -\sin\psi \\ 0 & 1 & 0\\ \sin\psi & 0 & \cos\psi \end{array}\right]\left[\begin{array}{ccc} 1 & 0 & 0\\ 0 & \cos\theta & \sin\theta \\ 0 & -\sin\theta & \cos\theta \end{array}\right]$$

Simplify (17) to get (18):

(18) $$C_{R}=\left[\begin{array}{ccc} \cos\psi \cos\gamma & \sin\theta \sin\psi \cos\gamma -\cos\theta \sin\gamma & -\cos\theta \sin\psi \cos\gamma -\sin\theta \sin\gamma \\ \cos\psi \sin\gamma & \sin\theta \sin\psi \sin\gamma +\cos\theta \cos\gamma & -\cos\theta \sin\psi \sin\gamma +\sin\theta \cos\gamma \\ \sin\psi & -\cos\theta \cos\psi & \cos\theta \cos\psi \end{array}\right]$$

Let the point on the rotor illuminated by measurement beam *II* be *p*. The coordinates of the motor before rotation are [*x_p_ y_p_ z_p_*]*^T^* and the coordinates after rotational transformation are [*X_p_ Y_p_ Z_p_*]*^T^*. The coordinates can be calculated by taking into account Equations (16)–(18) as follows:

(19) $$\left[\begin{array}{l} X_{p}\\ Y_{p}\\ Z_{p} \end{array}\right]=C_{R}\left[\begin{array}{l} x_{p}\\ y_{p}\\ z_{p} \end{array}\right]$$

Therefore, for any point on the rotor, as long as the three-axis angular velocity components *ω_x_*, *ω_y_*, and *ω_z_* of the rotor motion are measured by the position detection device, the position coordinates of any point on the rotor after motion can be obtained by performing the corresponding calculation with the above formula.

However, the gimbal lock phenomenon occurs when using this method, i.e., when using *o-xyz* as the basis and using the sequence of rotation around the *X*-axis, *Y*-axis, and *Z*-axis, once *±*90° is selected as the angle of the second rotation, it leads to the equivalence of the first and third rotations, and the whole rotational representation system is limited to the rotation around the vertical axis only, and a representation dimension is lost. The motors used in this experiment have less than 90° of deflection around the *X*-axis and *Y*-axis, and so the gimbal lock phenomenon has no effect on this experiment [23].

## 3. Experiment and Error Analysis

**Figure 5:**
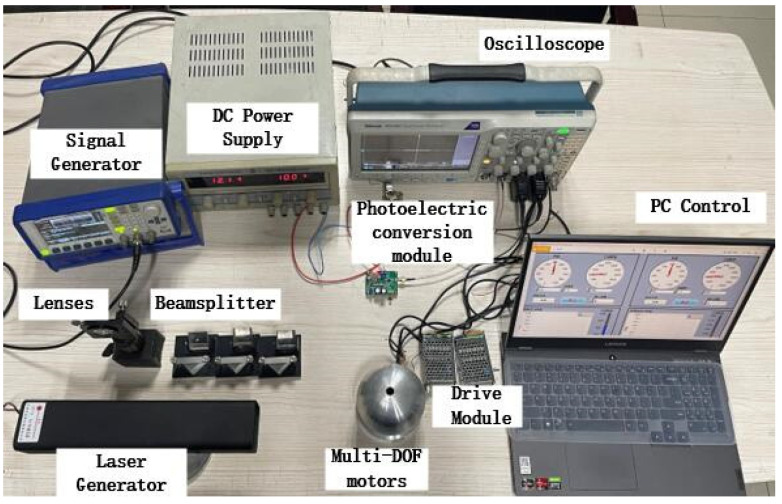
Measuring system and multi-DOF spherical motor.

The spherical rotor of a multi-DOF spherical motor is the main output component of this type of motor, which is capable of producing uniform rotation in multiple degrees of freedom. The experiment uses a piezoelectric driven multi-DOF spherical motor with a rated voltage of 12 V. The motor speed can be controlled by changing its voltage frequency to a maximum speed of 30 r/min, and its rotor outer diameter is measured to be 10 cm [24,25,26]. The experimental setup is shown in Figure 5 in the experiment, referring to Table 1, the theoretical maximum linear velocity around the *Z*-axis be achieved as follows:

(20) $$v=2\pi \times \frac{30}{60}\times 5\times 10^{-2}\approx 0.157\;m/s$$

During the experiment, the motor is idle and the measurement beam is irradiated at a point on the side of the spherical rotor, and a more stable observed output signal is obtained by adjusting the measurement beam irradiation position.

After adjusting the experimental equipment, the frequency of the motor input voltage is adjusted by the PC control system to change the motor speed, as shown in Table 1.

After adjusting the detection position so that *α* = 0°, adjust the motor to rotate at a constant speed, and verify the one-dimensional speed detection method through experiments, as shown in Figure 5. The signal generator controls the sampling frequency to generate a fixed frequency beam through the laser generator, and the laser is divided into a reference beam *I* and a measurement beam *II* when it passes through the beam splitter. The reference beam is scattered on the surface of the multi-DOF spherical motor, and then turned into parallel light by the lens, which is guided to converge on the receiving probe surface of the photoelectric conversion module. Finally, the optical signal is converted into an electric signal for processing by photoelectric conversion.

**Figure 6:**
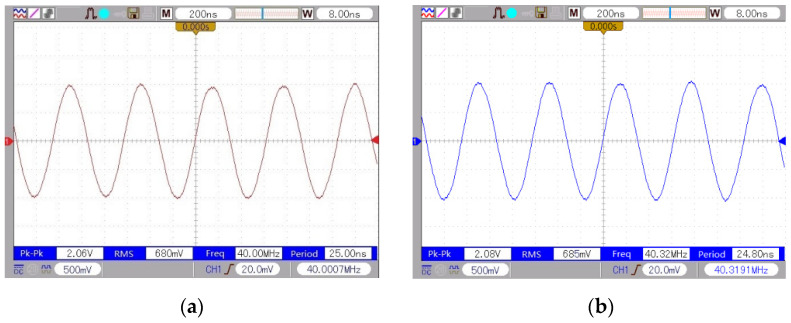
Frequency of the reference beam and the measurement beam: (**a**) reference beam frequency; (**b**) measurement of beam frequency.

In this experiment, eleven groups of motor data were measured at different rotational speeds, in which the group data were measured five times to obtain the average value to ensure the reliability of the data. Figure 6 shows the waveform of the reference beam and the measurement beam. The experimental data have good repeatability, smooth curve, and less burr, which means that the data are more stable.

As can be seen from Table 2, the relative error of measurement reaches a maximum of 2.56% at different rotational speeds, indicating that this laser speed measurement system can measure the motor speedwell and provide a reliable experimental basis for motor position detection.

**Figure 7:**
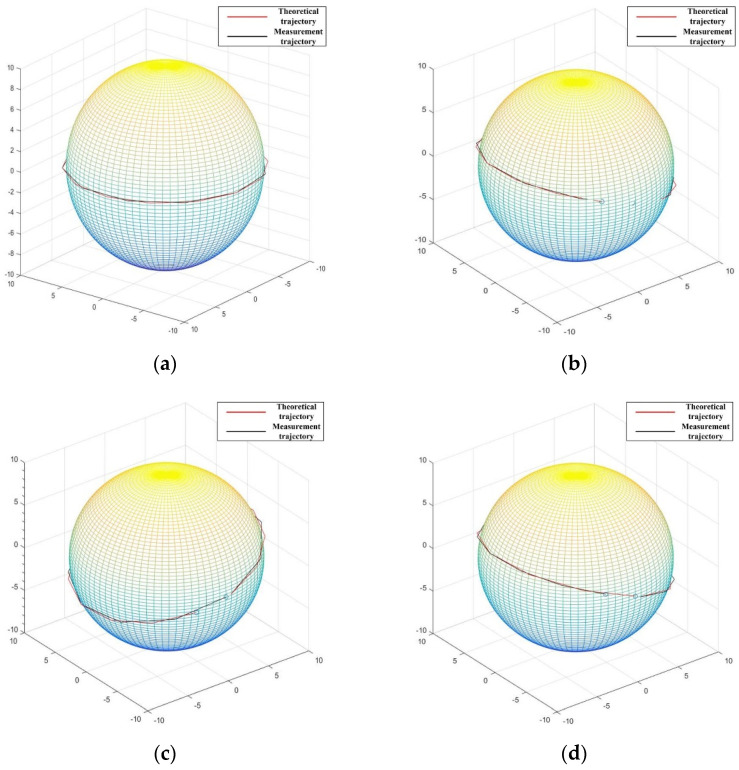
Measurement point trajectory: (**a**) only around *Z*-axis; (**b**) around *X*-axis and *Z*-axis; (**c**) around *Y*-axis and *Z*-axis; (**d**) around *X*-axis, *Y*-axis and *Z*-axis.

According to the correspondence in Table 1, the voltage frequency is adjusted to change the rotational speed to obtain several different trajectories, and the position of the experimental device is adjusted to measure the velocity components on the *X*, *Y*, and *Z* axes, and the rotor motion data collected by the photoelectric sensor is received and processed and calculated by the position detection algorithm. Finally, the measured trajectory of the detection point on the rotor during this experiment is obtained and the theoretical trajectory is derived based on the set rotational speed as a reference, as shown in Figure 7.

The motion trajectory of the measurement point obtained from the theoretical derivation data is used as the reference trajectory, and by comparing the position coordinates of the measurement point on the rotor at the same moment obtained from the laser detection experiment, the following error graph can be obtained, and the error calculation formula is:

(21) $$\{\begin{array}{c} error\_X=X_{1}-X_{2}\\ error\_Y=Y_{1}-Y_{2}\\ error\_Z=Z_{1}-Z_{2} \end{array}$$

where, *X*_1_, *Y*_1_, *Z*_1_ are the coordinates of the measured points in the motion trajectory derived from the experiments of the proposed laser position detection method, and *X*_2_, *Y*_2_, *Z*_2_ are the coordinates of the theoretically derived motion trajectory. 

**Figure 8:**
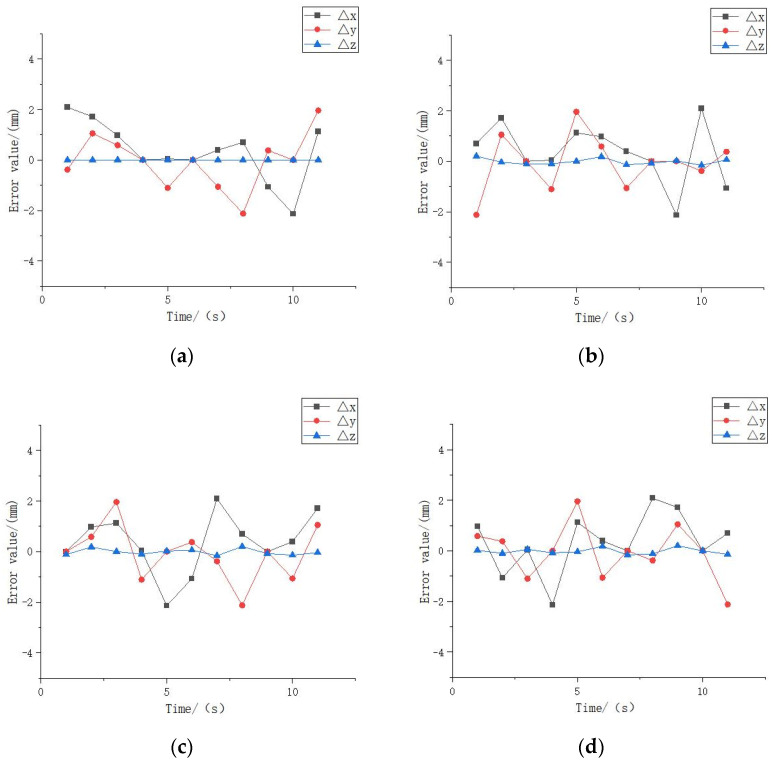
Position error: (**a**) error of Figure 7a; (**b**) error of Figure 7b; (**c**) error of Figure 7c; (**d**) error of Figure 7d.

The relative error curve can be derived from the above error calculation formula. As shown in Figure 8, the error of *Z* coordinate in the sampling point fluctuates around 0.1 mm~0.2 mm, which is much smaller compared with *X* coordinate and *Y* coordinate, mainly since the range of motion of this type of motor in Z-axis is relatively limited. the maximum forward and reverse error of *X* and *Y* axis is about 2 mm. This shows that this laser position detection method can reflect the motor position information better.

There are more sources of experimental errors. The hardware system itself has certain errors, such as laser optical path planning errors, measurement circuit design errors, etc. At the same time, the experimental environmental conditions constraints can also cause errors, such as interference from other light sources, the instability of the circuit connection, etc. Analysis of these errors can help the experiment to further improve the measurement accuracy.

The errors in the laser system mainly originate from the laser emitter, the optical mirror, and the photoelectric conversion device. Firstly, the light generated by the laser generator is not strictly monochromatic red light with a single frequency, which leads to errors in the frequency shift process when the Doppler effect is produced. Secondly, the optical mirror is not the ideal mirror, inevitably lost some scattered light, resulting in a certain deviation. At the same time, external interference light source is also a major cause of the impact of the optical path system. The optical conversion module is the main device to obtain the electrical signal, it is the main noise thermal noise, scattered grain noise, low-frequency noise, etc. These noises, after the amplifier amplification of the detection signal, producesome interference.

Thus, to reduce the error in the laser system, one should try to achieve a stable power supply for the laser generator, and preheat it in advance to make its output stable when in use; optimize the optical path, reduce the impact of scattering on light propagation, ensure the darkroom experimental environment; and reduce the impact of other light sources on the experiment.

The motor and the measurement circuit are also the main sources of error. In this experiment, as the motor speed increases, the motor vibration also increases, and the impact on the measurement will not be negligible, producing a large error. In addition, this measurement module needs to process the signal at about 40 MHz, so the electromagnetic interference between circuit components will also affect the measurement results and produce errors.

Therefore, fasteners should be added and the motor properly installed, choosing an appropriate speed to reduce the error brought by motor vibration on the measurement. One can try to use patch electronic components to reduce the impact of electromagnetic interference on the results.

## 4. Conclusions

This work uses a laser to measure the position information of a multi-DOF spherical motor. First, the feasibility of laser velocimetry is derived using the principle of the Doppler effect and verified through experiments, from which the velocity components of the multi-DOF spherical motor in three dimensions are derived to provide the basis for the next position detection part. On this basis, the theoretical formula of the position of the spherical rotor is derived and verified through experimental data to derive the position information of the multi-DOF. Based on this, the theoretical formula of the spherical rotor position is derived and verified by experimental data to derive the position information of the multi-degree of freedom motor detection points and compared with the theoretical value. The experimental results show that the accuracy of this method is high. Compared with the traditional position detection technique that requires attachment to the surface of the object to be measured, the laser detection technique uses a laser beam as the medium for information acquisition and transmission, and only requires the laser beam to irradiate the surface of the object to be measured, which is convenient to measure and does not affect the operating state of the motor to be measured and is more reliable. In the field of object vibration and position measurement, laser detection technology has a broad application prospect.

## Figures and Tables

**Table 1:** Motor frequency—speed reference.

Voltage Frequency/Hz	X-Axis Speed/(r/min)	Y-Axis Speed/(r/min)	Z-Axis Speed/(r/min)
42,250	30	16	16
42,750	22	13	12
43,250	14	7	7
43,750	9	5	5
44,250	4	2	2
44,750	3	3	2
45,500	3	2	2

**Table 2:** Speed measurement.

Motor Speed/(mm/s)	Frequency Deviation/MHz	Average Measurement Value/(mm/s)	Error
150	0.440	146.667	2.22%
140	0.429	143.000	2.14%
130	0.400	133.333	2.56%
120	0.364	121.333	1.11%
110	0.334	111.333	1.21%
100	0.302	100.667	0.67%
90	0.274	91.333	1.48%
80	0.242	80.667	0.83%
70	0.207	69.000	1.43%
60	0.178	59.333	1.11%
50	0.149	49.667	0.67%
